# Remarks on Dr. Porter's Doctrine Respecting Hydrocephalus

**Published:** 1819-09

**Authors:** D. Henderson


FOR THE LONDON MEDICAL AND PHYSICAL JOURNAL.

### *Remarks on Dr. Porter's Doctrine respecting Hydrocephalus.* By D. Henderson, Esq.

MR. Sutliffe presents us with a case of phrenitis in your Journal of last month, which he very properly combats, and happily subdues, by the application of leeches to the temples, aided by the free use of calomel and antimonial powder. But it is evident, from his own statement, that he considered it a case of hydrocephalus acutus; and for that reason he avows himself desirous to show that " the peculiar stress laid upon topical applications to the base of the cranium and nape of the neck, by Dr. Porter of Bristol," may be successfully dispensed with.

The author, whose practical instructions Mr. Sutliffe would subvert by the exhibition of his case with its result, has taken great pains to point out the distinctive marks of meningeal and of encephalic inflammatory condition. The case under consideration is a pure instance of the former, "pyrexia vehemens; dolor capitis; rubor facei; et occulorum lucis et soni intollerantia:" it does not present one feature of genuine acute hydrocephalus; and therefore, what is set forth in opposition to the practice recommended by Dr. Porter, rather serves as illustrative of the excellency of that physician's doctrine and practice. In phrenitis, abstract blood from the temples; in hydrocephalus acutus, from the nape. In the former all the branches of the external carotids are engaged; in the latter the seat of inflammation is the posterior, and most frequently to the exclusion of all phrenitic symptoms. But the diseases are sometimes mixed; then let the mode of attack be mixed. His pathology leads to these conclusions; and his distinctions are so far valuable, that they are not only theoretically right, but practically useful.

Mr. Sutliffe has shown his zeal for the advancement of practical knowledge by the publication of his case; and I hope these observations, which I feel due to the doctrine he would attack, may only serve to add a little reflection to any fact he may in future think worthy of public attention.

*Bristol; August* 3, 1819.

